# Accuracy and Precision of the COSMED K5 Portable Analyser

**DOI:** 10.3389/fphys.2018.01764

**Published:** 2018-12-21

**Authors:** Ismael Perez-Suarez, Marcos Martin-Rincon, Juan José Gonzalez-Henriquez, Chiara Fezzardi, Sergio Perez-Regalado, Victor Galvan-Alvarez, Julian W. Juan-Habib, David Morales-Alamo, Jose A. L. Calbet

**Affiliations:** ^1^Department of Physical Education, University of Las Palmas de Gran Canaria, Las Palmas, Spain; ^2^Research Institute in Biomedical and Health Sciences (IUIBS), University of Las Palmas de Gran Canaria, Las Palmas, Spain; ^3^Department of Mathematics, University of Las Palmas de Gran Canaria, Las Palmas, Spain

**Keywords:** oxygen uptake, ergometry exercise, metabolic cart, validity, reliability

## Abstract

The main aims of this study were to determine the accuracy of the portable metabolic cart K5 by comparison with a stationary metabolic cart (Vyntus CPX), to check on the validity of Vyntus CPX using a butane combustion test, and to assess the reliability of K5 during prolonged walks in the field. For validation, measurements were consecutively performed tests with both devices at rest and during submaximal exercise (bicycling) at low (60 W) and moderate intensities (130–160 W) in 16 volunteers. For the reliability study, 14 subjects were measured two times during prolonged walks (13 km, at 5 km/h), with the K5 set in mixing chamber (Mix) mode. Vyntus measured the stoichiometric RQ of butane combustion with high accuracy (error <1.6%) and precision (CV <0.5%), at VO_2_ values between 0.788 and 6.395 L/min. At rest and 60 W, there was good agreement between Vyntus and K5 (breath-by-breath, B×B) in VO_2_, VCO_2_, RER, and energy expenditure, while in Mix mode the K5 overestimated VO_2_ by 13.4 and 5.8%, respectively. Compared to Vyntus, at moderate intensity the K5 in B×B mode underestimated VO_2_, VCO_2,_ and energy expenditure by 6.6, 6.9, and 6.6%, respectively. However, at this intensity there was an excellent agreement between methods in RER and fat oxidation. In Mix mode, K5 overestimated VO_2_ by 5.8 and 4.8%, at 60 W and the higher intensity, respectively. The K5 had excellent reliability during the field tests. Total energy expenditure per Km was determined with a CV for repeated measurements of 4.5% (CI: 3.2–6.9%) and a concordance correlation coefficient of 0.91, similar to the variability in VO_2_. This high reproducibility was explained by the low variation of F_E_O_2_ measurements, which had a CV of 0.9% (CI: 0.7–1.5%) combined with a slightly greater variability of F_E_CO_2_, V_E_, VCO_2_, and RER. In conclusion, the K5 is an excellent portable metabolic cart which is almost as accurate as a state-of-art stationary metabolic cart, capable of measuring precisely energy expenditure in the field, showing a reliable performance during more than 2 h of continuous work. At high intensities, the mixing-chamber mode is more accurate than the B×B mode.

## Introduction

Nowadays, oxygen uptake (VO_2_), carbon dioxide production (VCO_2_), and substrate oxidation are mostly determined using stationary metabolic carts. For more specific measurements, particularly in the field, low-weight portable devices are more practical. Essentially, portable metabolic carts allow the measurement of the same variables as stationary metabolic carts with similar or slightly lower accuracy; however, the reliability of portable carts seems lower (Brehm et al., 2004; Schrack et al., 2010). Portable devices operate on a breath-by-breath basis (B×B), requiring the assessment of the tidal volume of each breath and the expiratory fractions of O_2_ (F_E_O_2_) and CO_2_ (F_E_CO_2_), which must be aligned in time for further calculations, by accounting for the respective delay times (Beaver et al., 1973; Noguchi et al., 1982).

The accuracy of portable and stationary metabolic carts working on a B×B mode, depends to a large extend on the exact determination of the delay times (Overstreet et al., 2017). Most modern stationary metabolic carts have remarkably reduced delay times, and some, like the Vyntus CPX (Jaeger-CareFusion) have delay times for O_2_ and CO_2_ analysis almost matching mass spectrometers (Clemensen et al., 1994). Short delay times are necessary to avoid errors in B×B analysis at high respiratory frequencies, i.e., at high exercise intensities (Overstreet et al., 2017). Underestimation of delay time results in overestimations of F_E_O_2_, resulting in underestimation of VO_2_ (Beaver et al., 1973; Noguchi et al., 1982; Hughson et al., 1991). Conversely, overestimation of delay time results in underestimation of O_2_ and overestimation of VO_2_ (Beaver et al., 1973; Noguchi et al., 1982; Hughson et al., 1991). This limitation can be overcome by the use of a mixing chamber, were a representative micro-sample of each breath is temporally stored and mixed with previous samples before analysis (Overstreet et al., 2017). A new portable metabolic cart (COSMED K5) has been marketed with the option of measuring using either B×B or using a micro-proportional sampling averaging method. However, the accuracy and reliability of the COSMED K5 remains unknown.

Therefore, the purpose of this study was to determine the accuracy of COSMED K5 by comparison with a stationary metabolic cart at rest and during submaximal cycling exercise. Another aim was to measure the reliability of K5 for the assessment of energy expenditure during prolonged walks in the field. Walking rather than running was chosen to avoid potential artifacts due to mask displacement and to reduce thermal strain, so the subjects will not need to stop to remove the mask and drink. Since the validity of the Vyntus CPX remains unknown, a final aim was to determine the validity of Vyntus CPX using a butane combustion test.

## Materials and Methods

### Participants

Two different studies were carried out to assess validity and reliability on two different groups separately. Sixteen Caucasian physically active volunteers (3 women and 13 men), between 19 and 32 years old, volunteered to participate in the validation study (Table 1). Another fourteen Caucasian physically active volunteers (3 women and 11 men), between 20 and 43 years old, agreed to participate in the reliability study (Table 1). All volunteers provided their written consent after being informed about the risks and benefits of the study, which was approved by the ethical committee of the University Hospital Dr. Negrin (Ref. 140187). All subjects were requested not to exercise and to refrain from drinking alcohol and beverages containing caffeine or taurine during the 48 h preceding the tests, as well as to eat a similar dinner the night before the tests.

**Table 1:** Descriptive characteristics of K5 validation subjects (resting metabolic rate and exercise tests).

	Validation study (*n* = 16)		Reliability study (*n* = 14)	
	
Parameter	Mean ± SD	Range	Mean ± SD	Range
Age (years)	23.3 ± 3.4	19.0 – 32.0	25.4 ± 6.5	20.9 – 43.1
Weight (kg)	72.3 ± 10.3	61.0 – 96.3	70.6 ± 10.6	55.9 – 89.3
Height (cm)	175.4 ± 7.4	162.2 – 185.0	174.2 ± 7.6	163.8 – 188.3
BMI (kg/m^2^)	23.4 ± 2.5	20.7 – 29.1	23.1 ± 2.2	19.7 – 27.2

*Values are means ± standard deviation (SD). BMI, body mass index.*

### General Procedures

Subjects reported to the laboratory between 7:00 and 9:30 A.M., following a 12-h overnight fast. Upon arrival, their body weight and height were measured to the nearest 0.1 kg and 0.1 cm, respectively. Measurements were performed while subjects wore light clothes and no shoes using a balance scale (Seca, Hamburg, Germany) calibrated using certified calibration masses of class M1 (Scheck, Germany).

The validity of the COSMED K5 was determined by comparison with the Vyntus CPX (Jaeger-CareFusion, Höchberg, Germany) both at rest and during submaximal exercise on a cycle ergometer (Corival, Lode, Netherlands). Both metabolic carts were equipped with relatively new (less than 3 months old) oxygen fuel cells). Before the measurements both devices were warmed up for a minimum of 15 min calibrated with high-grade calibration gases provided by the manufacturers and by pumping gas with a 3 L calibration syringe through the flow meters, following the recommendations of the manufacturers. Mask size was individually fitted prior to the first test and the same size was maintained for subsequent trials.

#### Validation Study

For this purpose, subjects were instrumented with the Vyntus or COSMED facemask, in random order. The Vyntus was operated BxB and the COSMED K5 in BxB and mixing chamber modes. After arrival, subjects rested awake in the supine position during the next 60 min on a comfortable laboratory stretcher provided with a pillow, while their resting metabolic rate (RMR) was determined with both metabolic carts. Subjects were instructed to lie motionless and avoid talking during the measurement, which was carried out in a well-ventilated room while maintaining a quiet environment. The ergospirometric values recorded between the 20^th^ and 30^th^ min were averaged and used as representative of the metabolic cart tested first. During the following 5 min the device was changed in case the measurements started with the Vyntus, or switched from B×B to mixing chamber if the first device tested was the COSMED K5 and then to Vyntus. Thus, 10 min periods were collected with each device, with the first measurements starting after at least 20 min of comfortable rest in the supine position. A new full calibration (gas and flow) was done before switching systems.

Then the volunteers performed two bouts of exercise on the cycle ergometer at 60 W for 10 min, followed by 6 min at 130 W (women) or 160 W (men), keeping the pedaling rate at 80 rpm. These intensities were chosen to test the K5 at a low exercise intensity eliciting VO_2_ values similar to those observed during brisk walking (i.e., ∼6 km/h) (van der Walt and Wyndham, 1973), as well to a moderate exercise intensity eliciting a VO_2_ value close to the VO_2max_ observed in sedentary populations (Edvardsen et al., 2013). To reduce potential carry-over effects when changing from one metabolic cart to the other and to allow the assessment of substrate oxidation by indirect calorimetry, the highest load was chosen to elicit RER values close to 1.00. This sequence was repeated in random order while data were collected with the Vyntus, the COSMED K5 set on the B×B and mixing-chamber modes allowing a 20-min rest period between the bouts. Seat and handlebar adjustments were fit to the subject specifications and remained unchanged during all bouts.

#### Reliability Study

The reliability of the COSMED K5 was assessed by measuring ergospirometric variables and the energy expenditure during prolonged walks (13 km), which were repeated two times at least 4 days apart, with the device set in the mixing chamber mode. These tests were performed at the same time of day on non-raining days, and subjects were requested to wear similar clothing and the same walking shoes for the two trials. The walking route was relatively flat; including land and concrete sidewalks sections. The walking speed was maintained close to 5 km/h, using a Garmin Forerunner 210 (Garmin International Inc., Olathe, KS, United States) with GPS. The heart rate was measured with a Garmin Sensor connected to the K5. All subjects were weighted immediately before and after walking (SECA 869, Hamburg, Germany) while wearing all equipment and clothes. The scale was calibrated with certified calibration masses of class M1.

#### Energy Expenditure

Energy expenditure was calculated from VO_2_ and VCO_2_ data, assuming a negligible contribution of protein oxidation as previously reported (Peronnet and Massicotte, 1991).

### Validation of Vyntus CPX by Burning Butane

The combustion of butane (2 C_4_H_10_ + 13 O_2_ → 8 CO_2_ + 10 H_2_O + heat) has a respiratory quotient of 0.615 (8/13). Thus, we built an air-tight Plexiglas box (70 cm × 50 cm × 50 cm) with two 3.05 cm circular openings. One opening was connected to a 3-L calibration syringe (Hans Rudolf, Shawnee, KS) and in the other the turbine of the Vyntus with the sample lines for gas analysis was placed. Inside the Plexiglas box, a small butane burner was ignited while a 5 liter stainless steel cooking pot was placed on top filled with 0.5 L of water and ice up to the top, to dissipate the heat generated by the butane combustion. Three different burning intensities were used to simulate low, medium and very high exercise intensity. Within 5 s after the ignition of the butane burner, air was pumped in and out continuously using the calibration syringe, adjusting the stroke rate between 15 and 80 strokes/min until an almost “steady state” was reached, as reflected by the F_E_O_2_ and F_E_CO_2_. The pumping rate was then maintained for 2 min. Butane combustion data were averaged every 10 s to determine the precision of the metabolic cart, and the coefficient of variation (CV) determined for a set of 5–6 consecutive averages with steady ventilation. The ventilation was considered steady when consecutive values differ by less than 10%. Some variability in ventilation was unavoidable since the air was pumped manually, although always by the same operator.

### Statistical Analysis

Data are reported as the mean (±*SD*), unless otherwise stated. Values were checked for normal distribution using the Shapiro-Wilks test. The agreement between methods was assessed by determining the bias in absolute values and as a percentage of the measured value with the reference method (Vyntus) and the corresponding limit of agreement upper limit of agreement (ULA) = bias + 1.96 × *SD*; lower limit of agreement (LLA) = bias – 1.96 × *SD*. In addition, the agreement between methods was further examined by determining the Concordance Correlation Coefficient (CCC; Lin, 1989). The accuracy was evaluated by determining the differences between the mean values obtained with each method using Student *t*-test. The reliability of COSMED K5 measurements was assessed by determining the CV as described by Forkman (2007). Finally, the relationship between VO_2_ and VCO_2_ during butane combustion at different rates was assessed by the correlation coefficient of Pearson using 10-s averaged data. A *P*-value ≤ 0.05 was considered to be statistically significant. All statistical analyses were performed using IBM SPSS v.21.0 for Mac Computers (IBM, New York, United States).

## Results

### *In vitro* Validation of Vyntus

We used three different levels of combustion to simulate low, medium, and very high exercise intensities (Supplementary Table 1). The corresponding VO_2_ values were 0.788, 1.314, and 6.395 L/min and the associated RQ values 0.624, 0.608, and 0.619, respectively. The RQ deviation from the theoretical value for the combustion of butane (0.615) were 1.5, -1.1, and 0.7%, respectively. The CV of RQ for 5–6 consecutive 10 s averaged-intervals with similar ventilation was always below 0.5%. VO_2_ during butane combustion was closely related to VCO_2_ (*r* = 0.9999).

### Accuracy and Precision

**FIGURE 1:**
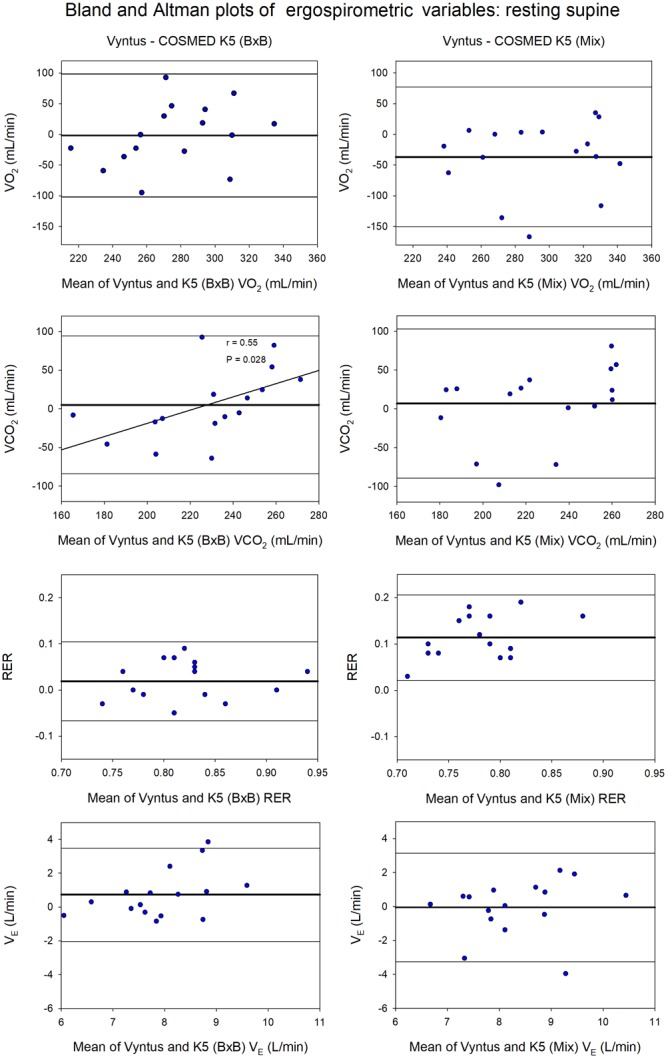
Agreement between Vyntus (used as a reference stationary metabolic cart) and COSMED K5 operated Breath-by-Breath (B×B) and in Mixing Chamber (Mix) modes during resting measurements for oxygen uptake (VO_2_), carbon dioxide production (VCO_2_), respiratory exchange ratio (RER) and pulmonary ventilation (V_E_). *N* = 16.

**FIGURE 2:**
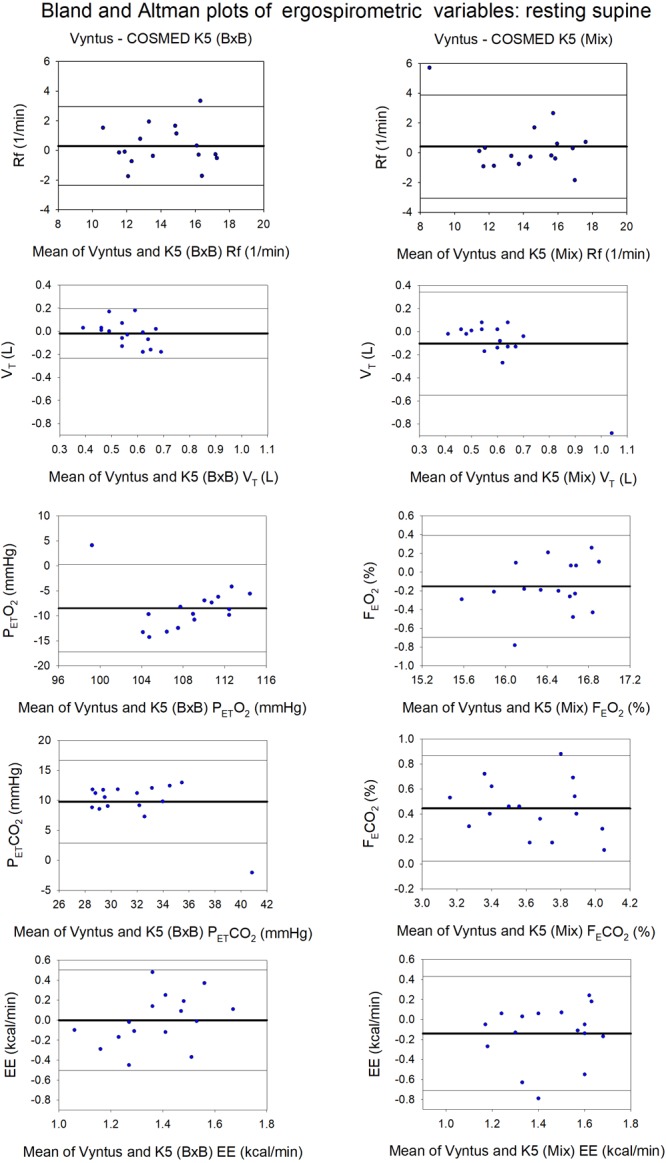
Agreement between Vyntus (used as a reference stationary metabolic cart) and COSMED K5 operated Breath-by-Breath (B×B) and in Mixing Chamber (Mix) modes during resting measurements for respiratory frequency (Rf), tidal volume (V_T_), end-tidal O_2_ pressure (P_ET_O_2_), end-tidal CO_2_ pressure (P_ET_CO_2_), and energy expenditure (EE). *N* = 16.

**FIGURE 3:**
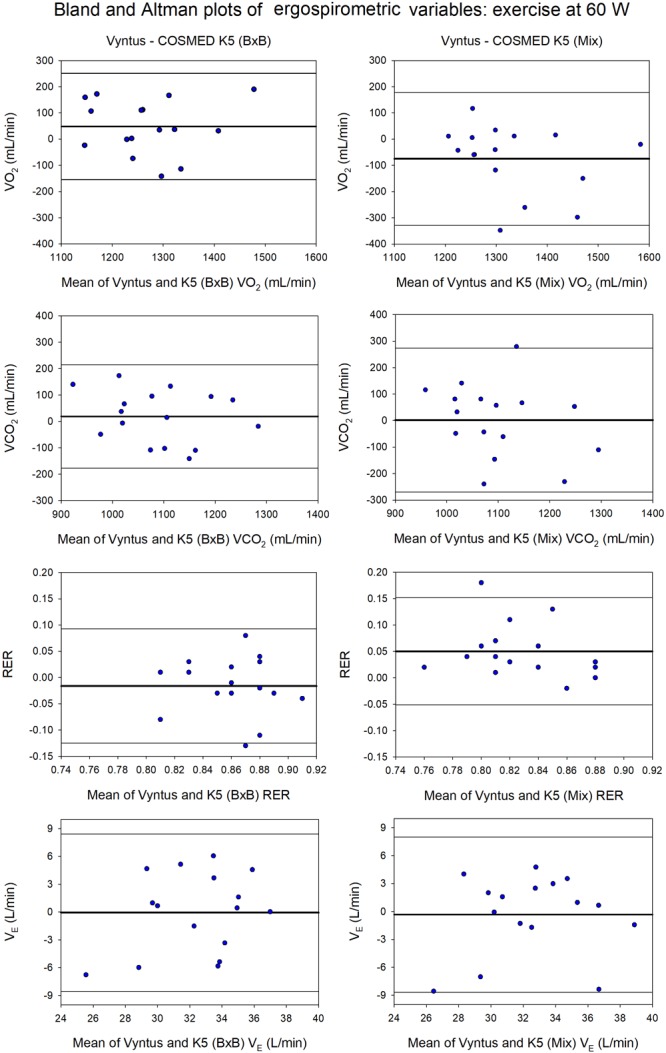
Agreement between Vyntus (used as a reference stationary metabolic cart) and COSMED K5 operated Breath-by-Breath (B×B) and in Mixing Chamber (Mix) modes during low-intensity exercise (60W) for VO_2_, VCO_2_, RER, and pulmonary ventilation (V_E_). *N* = 16.

**FIGURE 4:**
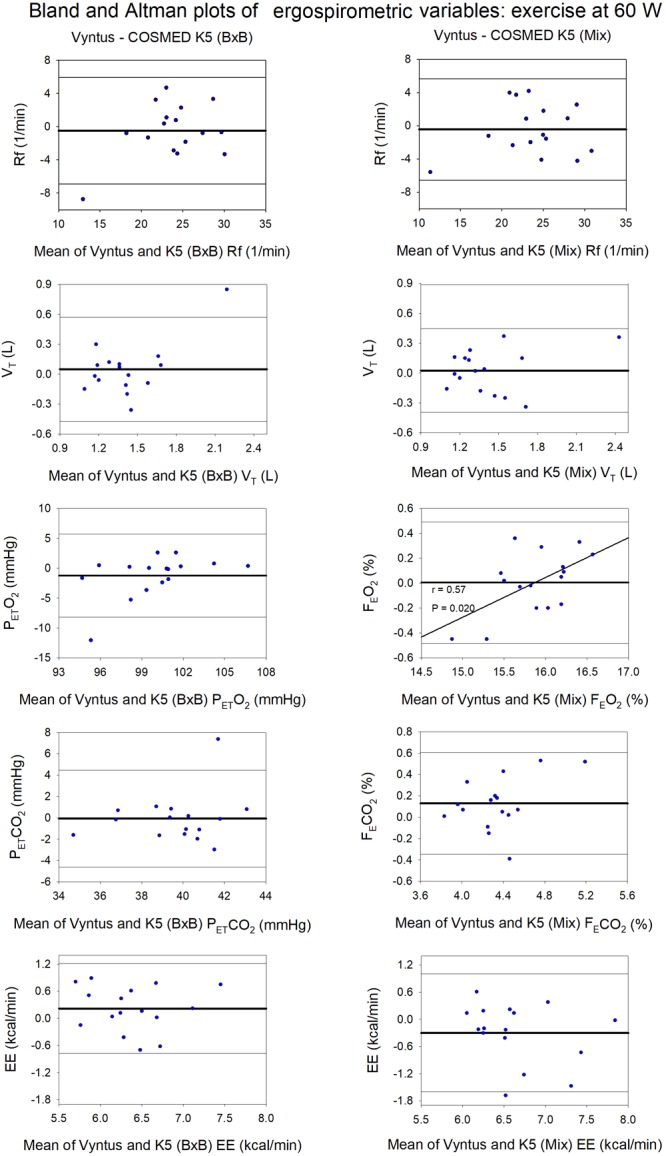
Agreement between Vyntus (used as a reference stationary metabolic cart) and COSMED K5 operated Breath-by-Breath (B×B) and in Mixing Chamber (Mix) modes during low-intensity exercise for respiratory frequency (Rf), tidal volume (V_T_), end-tidal O_2_ pressure (P_ET_O_2_), expiratory O_2_ fraction (F_E_O_2_), end-tidal CO_2_ pressure (P_ET_CO_2_), expiratory CO_2_ fraction (F_E_CO_2_), and energy expenditure (EE). *N* = 16.

During measurements at rest and 60 W Vyntus and COSMED K5 (B×B) reported similar mean VO_2_, VCO_2_, RER, and energy expenditure values (Supplementary Tables 2, 3 and Figures 1–4). Nevertheless, compared to Vyntus, COSMED K5 operated in mixing chamber mode (Mix) overestimated VO_2_ by 13.4 and 5.8%, at rest and 60 W, respectively (Supplementary Table 2). Both Vyntus and COSMED K5 (Mix) reported similar V_E_ and VCO_2_ values. Consequently, COSMED K5 (Mix) underestimated RER by 13.8 and 5.8%, at rest and at 60 W, respectively.

**FIGURE 5:**
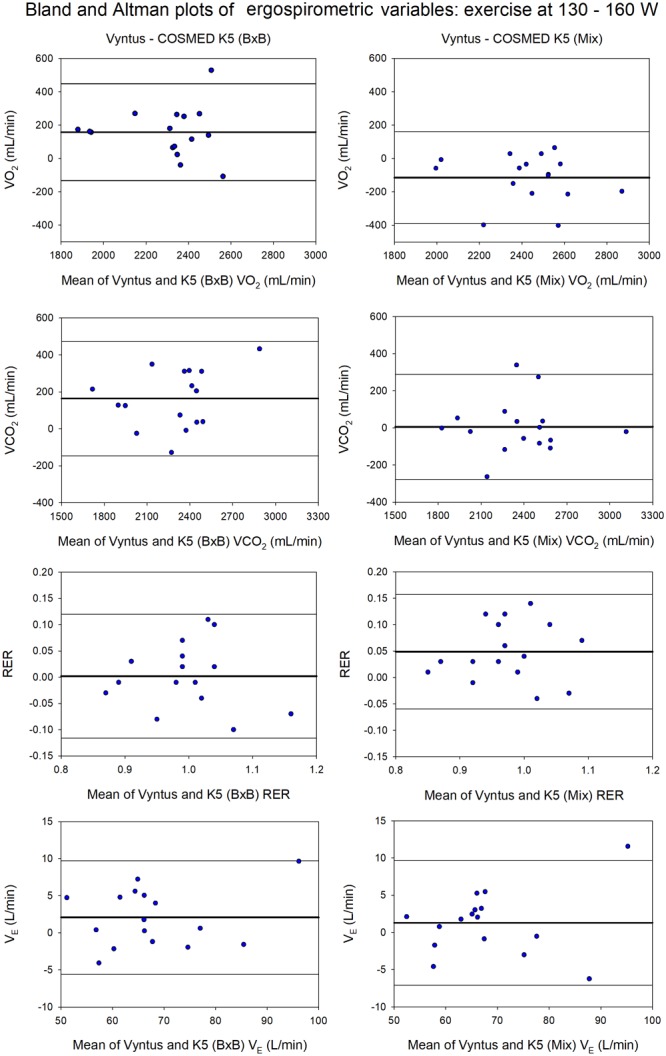
Agreement between Vyntus (used as a reference stationary metabolic cart) and COSMED K5 operated Breath-by-Breath (B×B) and in Mixing Chamber (Mix) modes during moderate-intensity exercise (130–160 W) for oxygen uptake (VO_2_), carbon dioxide production (VCO_2_), respiratory exchange ratio (RER) and pulmonary ventilation (V_E_). *N* = 16.

**FIGURE 6:**
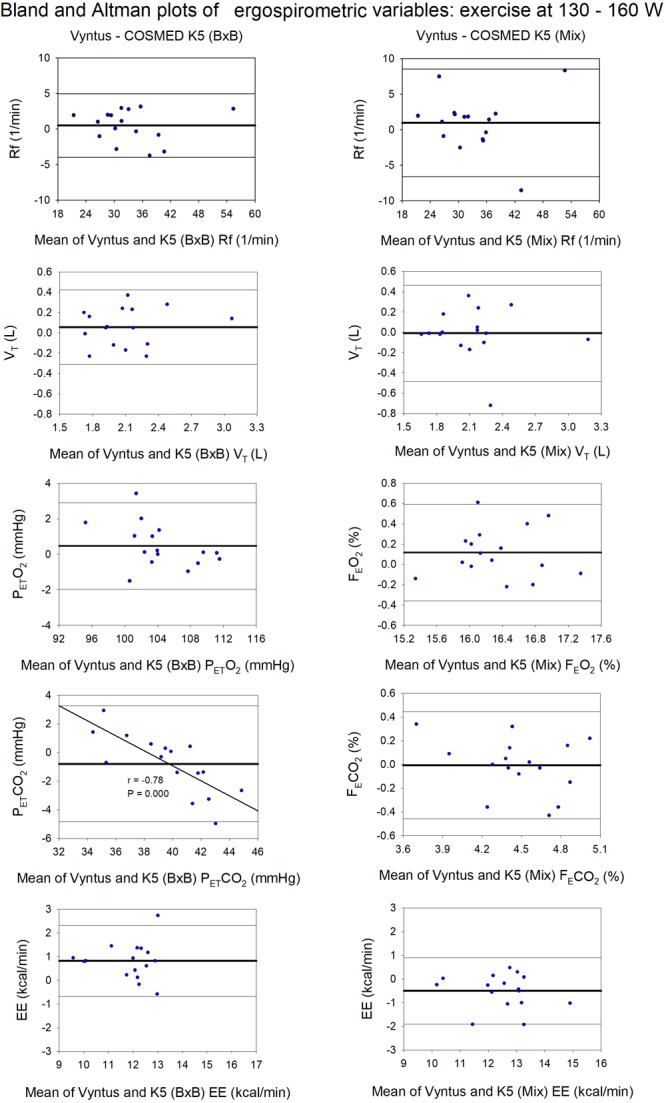
Agreement between Vyntus (used as a reference stationary metabolic cart) and COSMED K5 operated Breath-by-Breath (B×B) and in Mixing Chamber (Mix) modes during moderate-intensity (130–160 W) exercise for respiratory frequency (Rf), tidal volume (V_T_), end-tidal O_2_ pressure (P_ET_O_2_), expiratory O_2_ fraction (F_E_O_2_), end-tidal CO_2_ pressure (P_ET_CO_2_), expiratory CO_2_ fraction (F_E_CO_2_), and energy expenditure (EE). *N* = 16.

At the highest intensity (130 W and 160 W in women and men, respectively) COSMED K5 (B×B) underestimated VO_2_ and VCO_2_ by 6.6 and 6.9%, respectively, due to a 3.0% underestimation of V_E_ (B×B) combined with 1.8% F_E_O_2_ overestimation and 5.1% F_E_CO_2_ underestimation by COSMED K5 (B×B) (Supplementary Table 3 and Figures 5, 6). This resulted in a 6.6% underestimation of energy expenditure by COSMED K5 (B×B). Nevertheless, there was an excellent agreement between methods in RER and fat oxidation, while COSMED underestimated carbohydrate oxidation by 6.9% (Supplementary Table 3).

COSMED K5 (Mix) overestimated VO_2_ during exercise by 5.8 and 4.8%, at 60 W and the highest intensity, respectively. Consequently, COSMED K5 (Mix) overestimated energy expenditure by 4.1%. Since VCO_2_ was accurately measured during exercise by COSMED K5 (Mix), COSMED K5 (Mix) underestimated the RER by 5.8 and 4.8%, at 60 W and the highest intensity, respectively, causing a marked overestimation of fat oxidation (Supplementary Table 3).

### COSMED K5 Breath-by-Breath Compared With the Mixing Chamber Mode

Compared to B×B, the mixing chamber mode overestimated VO_2_ by 12.7% at rest (Supplementary Table 4). This bias in the assessment of resting VO_2_ is mostly explained by a 10.4% V_E_ overestimation in Mix mode. However, VCO_2_ was accurately measured due to the compensation of the overestimation of V_E_ by an underestimation of F_E_CO_2_ in Mix mode. Consequently, the RER was underestimated by 11.8% and energy expenditure by 10.1% in Mix mode.

During exercise the Mix mode the K5 overestimated VO_2_ by 9.9 and 12.3% at 60 W and the highest load, respectively (Supplementary Table 5). This was explained by a 2.2 and 2.4% F_E_O_2_ underestimation, respectively. At 60 W, there was no bias in the assessment of VCO_2_, but it was overestimated by 7.1% at the highest intensity by the Mix mode, resulting in 7.5 and 4.7% underestimation of the RER at 60 W and the highest load, respectively. Consequently, the Mix mode overestimated energy expenditure by 8.3 and 11.5% at 60 W and highest load, respectively.

### Reliability

The COSMED K5 had excellent reliability during the field tests (Supplementary Table 6), despite small changes in walking speed, starting weight, and environmental conditions (Supplementary Table 7). Total energy expenditure per Km was determined with a CV for repeated measurements of 4.5% (CI: 3.2–6.9%) and a CCC of 0.91, similar to the variability of VO_2_. This high reproducibility was explained by the low variation of F_E_O_2_ measurements, which had a CV of 0.9% (CI: 0.7–1.5%) combined with a slightly greater variability of F_E_CO_2_, V_E_, VCO_2_, and RER.

## Discussion

In the present investigation, the Vyntus CareFusion metabolic cart was used as the reference method. Although Vyntus is a relatively new metabolic cart and no data about its validity against the Douglas bag method is available, its precedent Oxycon Pro was proven to be valid (Foss and Hallen, 2005). Moreover, this has been confirmed for the Vyntus when cross-calibrated against the Vmax29 (Carlomagno et al., 2015). Our butane combustion tests have also shown that the Vyntus CPX can measure the stoichiometric respiratory quotient of butane combustion at low and very high O_2_ flow with unprecedented accuracy and precision. Besides, the present investigation shows that the new COSMED K5 portable ergospirometer is an instrument that reproduces accurately the values obtained with the Vyntus metabolic cart at rest and during low-intensity exercise when operated in the B×B mode. We have also demonstrated that the COSMED K5 is highly precise at low-intensity exercise even during more than 2 h of continuous operation.

In the B×B mode, the COSMED K5 measures resting and low-intensity exercise (approximately 5 METs) VO_2_, VCO_2_, RER, and energy expenditure with an accuracy similar to that of the Vyntus metabolic cart, which has been recently marketed by the CareFusion company as a new metabolic cart based on its predecessors JAEGER Oxycon Pro and SensorMedics^TM^ Vmax^TM^ Encore. Nevertheless, at a higher exercise intensity equivalent to approximately 9 METs, COSMED K5 underestimates by 6–7% VO_2_ and VCO_2_, mostly due to underestimation of V_E_ and a small bias in the assessment of F_E_O_2_ and F_E_CO_2_.

Inclusion of a mixing chamber mode is the main innovation incorporated in the COSMED K5 compared to its predecessor (K4B^2^). In a stationary metabolic cart, the addition of a mixing chamber allows to mix the expiratory gases from 3–6 breaths (depending on the size of the mixing chamber and the tidal volume). This allows for a more accurate assessment of the F_E_O_2_ and F_E_CO_2_. Nevertheless, in a portable device the size of the mixing chamber is limited and only a fraction (proportional micro-sample) of the tidal volume is aspirated into the chamber for mixing with precedent breaths. However, our results demonstrate that at rest and during low exercise intensity, the mixing chamber is less accurate than the B×B mode. Since there was a good agreement in V_E_ and VCO_2_ between the mixing chamber and the B×B modes of the COSMED K5, the divergence here observed in VO_2_ between these two modes is likely due to the impact that the assumption of a fixed F_I_O_2_ (0.209) has on the calculation of VO_2_. At higher exercise intensities, the tidal volume increases and the impact of the facemask dead space on the F_I_O_2_ is reduced, i.e., the actual F_I_O_2_ becomes closer to the assumed by the COSMED K5 (0.209) reducing the bias in the assessment of VO_2_ at the highest exercise intensity. Our data also demonstrate that in the mixing chamber mode, the COSMED K5 measured the F_E_O_2_ with similar accuracy as Vyntus, while in the B×B mode it overestimated the F_E_O_2_ obtained with the Vyntus. Thus, when the aim of the study is to measure VO_2_ or substrate oxidation at high exercise intensities the mixing chamber mode is preferable. In contrast, measuring the resting RER with the COSMED K5 set in mixing chamber mode yields RER values close to 0.70, a value that is too low for subjects that have been fasting only overnight (Compher et al., 2006). Thus, at rest and during low intensity exercise the B×B mode is more accurate.

Our data also indicate that the COSMED K5 performs at least as well as its predecessor COSMED K4B^2^ (Doyon et al., 2001; McLaughlin et al., 2001; Pinnington et al., 2001; Eisenmann et al., 2003; Duffield et al., 2004; Mc Naughton et al., 2005), while the addition of the mixing chamber mode allows more accurate assessments at high-intensity exercise.

### Precision of COSMED K5

The precision of COSMED K5 was determined by using the mixing chamber mode during prolonged low intensity exercise in the field. Our data demonstrate that the COSMED K5 can assess VO_2_ and energy expenditure during prolonged walking with a CV below 5%, i.e., performing at a similar level of precision as stationary metabolic carts (Carter and Jeukendrup, 2002; Crouter et al., 2006). It should be taken into consideration that this low CV was obtained despite small changes in body weight, environmental conditions, walking speed, and weight loss during the walks. Thus, even lower CVs may be achievable with the K5 under better controlled environmental conditions as during test carried out in laboratories. This level of precision exceeds that reported for its predecessor the K4B^2^ for repeated walking measurements in separate days during treadmill walking (Darter et al., 2013; Howe et al., 2014) as well as during incremental exercise (Schrack et al., 2010; Brisswalter and Tartaruga, 2014).

### Accuracy and Precision of Vyntus CPX (Butane Combustion Tests)

Combustion of fuels like ethanol (Cooper et al., 1991; Rising et al., 2015), methanol (Damask et al., 1982; Miodownik et al., 1998), propane (Melanson et al., 2010; Rising et al., 2015; Ismail, 2017), and butane (Nunn et al., 1989; Joosten et al., 2000) are considered gold-standard methods to validate indirect calorimeters. A common feature of precedent propane and butane combustion studies has been the use of only low combustion rates. In the present investigation we have used a similar methodology but using a broad range of combustion rates eliciting VO_2_ values spanning across the physiological VO_2_ values reachable by humans with varied fitness levels. In addition, our simulation generated respiratory variables mostly within the physiological range for humans. Importantly, our data indicate that the Vyntus CPX metabolic cart is exceptionally accurate and precise measuring the stoichiometric RQ of butane combustion. This is only possible when VO_2_ and VCO_2_ are computed correctly from the inspired and expired O_2_ and CO_2_ fractions, even at high breathing frequencies (highest butane combustion rate) and despite differences in incurrent and excurrent airstreams (see Supplementary Table 1).

In summary, our data indicate that the COSMED K5 is an excellent portable metabolic cart which is almost as accurate as a state-of-art stationary metabolic cart, capable of measuring precisely energy expenditure in the field and showing a reliable performance during more than 2 h of continuous work. The mixing-chamber option allows for more accurate assessment of VO_2_ during exercise at relatively higher intensities, but is less accurate at low exercise intensities, and should not be used to measure resting energy expenditure.

## Author Contributions

JC, DM-A, and IP-S conceived and designed the experiments. IP-S, MM-R, CF, SP-R, VG-A, JJ-H, and DM-A performed the pre-testing, experimental preparation, and data collection. IP-S, SP-R, CF, and JC analyzed the data. IP-S and JC wrote the first draft of the manuscript. All co-authors edited and proofread the manuscript and approved the final version.

## Conflict of Interest Statement

The authors declare that the research was conducted in the absence of any commercial or financial relationships that could be construed as a potential conflict of interest.
