# Supplementary material for: Accuracy and Precision of the COSMED K5 Portable Analyser
Source: Front Physiol. 2018 Dec 21;9:1764. doi: 10.3389/fphys.2018.01764 (PMC6308190; doi:10.3389/fphys.2018.01764)
Supplement: Supplementary file 1 [file Table_1.docx]

| **Table 1.** Butane combustion assessed by Vyntus CPX | | | | | | | | | | | | | | |
| --- | --- | --- | --- | --- | --- | --- | --- | --- | --- | --- | --- | --- | --- | --- |
| Simulated Exercise intensity | VO_2_ | VCO_2_ | RQ | F_I_O_2_ | F_I_CO_2_ | F_E_O_2_ | F_E_CO_2_ | P_ET_O_2_ | P_ET_CO_2_ | V_E_ | VT_ex_ | VT_in_ | SR |  |
|  | mL/min | mL/min |  | % | % | % | % | mmHg | mmHg | L/min | L | L | Strokes/min |  |
|  |  |  |  |  |  |  |  |  |  |  |  |  |  |  |
| Low | 788 | 492 | 0.624 | 20.25 | 0.44 | 17.82 | 2.09 | 127 | 14 | 37 | 1755 | 1757 | 21 |  |
| Moderate | 1314 | 798 | 0.608 | 20.60 | 0.22 | 18.09 | 1.89 | 129 | 12 | 60 | 1724 | 1613 | 35 |  |
| Very high | 6395 | 3962 | 0.620 | 20.77 | 0.10 | 15.85 | 3.41 | 111 | 24 | 150 | 1964 | 1714 | 77 |  |
| VO_2_, oxygen uptake; VCO_2_, carbon dioxide production; RQ, respiratory quotient, F_I_O_2_, inspiratory O_2_ fraction; F_I_CO_2_, inspiratory CO_2_ fraction; F_E_O_2_, expiratory O_2_ fraction; F_E_CO_2_, expiratory CO_2_ fraction; P_ET_O_2_, end-tidal O_2_ pressure; P_ET_CO_2_, end-tidal CO_2_ pressure; V_E_, ventilation; VT_ex_, tidal volume excurrent airstream; VT_in_, tidal volume incurrent airstream; SR, stroke frequency with the calibration syringe. | | | | | | | | | | | | | | |
