# Supplementary material for: Accuracy and Precision of the COSMED K5 Portable Analyser
Source: Front Physiol. 2018 Dec 21;9:1764. doi: 10.3389/fphys.2018.01764 (PMC6308190; doi:10.3389/fphys.2018.01764)
Supplement: Supplementary file 2 [file Table_2.docx]

| **Table 2.** Accuracy of COSMED K5 (Breath by Breath) compared to Vyntus during resting measurements | | | | | | | | | | | | |
| --- | --- | --- | --- | --- | --- | --- | --- | --- | --- | --- | --- | --- |
|  | **Vyntus** | | | **COSMED K5 (BxB)** | | | **Mean differences** | **Limits of agreement** | | | **T-Test P-Value** | **CCC** |
|  | **Mean** | **±** | **SD** | **Mean** | **±** | **SD** |  | **Lower** | - | **Upper** |  |  |
| VO_2_ (mL/min) | 275 | ± | 47 | 277 | ± | 33 | -1.58 | -101.97 | - | 98.82 | 0.904 | 0.21 |
| VCO_2_ (mL/min) | 231 | ± | 46 | 225 | ± | 25 | 5.26 | -83.96 | - | 94.47 | 0.651 | 0.24 |
| RER | 0.83 | ± | 0.06 | 0.82 | ± | 0.05 | 0.02 | -0.07 | - | 0.10 | 0.118 | 0.66 |
| Rf (1/min) | 14.4 | ± | 2.3 | 14.1 | ± | 2.3 | 0.30 | -2.36 | - | 2.97 | 0.389 | 0.82 |
| V_T_ (L) | 0.55 | ± | 0.08 | 0.57 | ± | 0.12 | -0.02 | -0.23 | - | 0.20 | 0.517 | 0.43 |
| V_E_ (L/min) | 8.3 | ± | 1.4 | 7.6 | ± | 0.8 | 0.73 | -2.03 | - | 3.49 | 0.056 | 0.20 |
| V_E_/VO_2_ | 30.5 | ± | 4.1 | 27.5 | ± | 2.2 | 3.00 | -3.09 | - | 9.09 | 0.002 | 0.39 |
| V_E_/VCO_2_ | 36.5 | ± | 4.5 | 33.7 | ± | 2.9 | 2.74 | -4.14 | - | 9.62 | 0.007 | 0.46 |
| F_I_O_2_ (%) | 20.3 | ± | 0.3 | 20.9 | ± | 0.0 | -0.60 | -1.23 | - | 0.03 | 0.000 | 0.00 |
| F_E_O_2_ (%) | 16.36 | ± | 0.43 | 16.51 | ± | 0.33 | -0.16 | -0.61 | - | 0.30 | 0.017 | 0.75 |
| F_I_CO_2_ (%) | 0.47 | ± | 0.24 | 0.05 | ± | 0.01 | 0.41 | -0.05 | - | 0.88 | 0.000 | 0.01 |
| F_E_CO_2_ (%) | 3.86 | ± | 0.27 | 3.77 | ± | 0.32 | 0.09 | -0.22 | - | 0.40 | 0.039 | 0.82 |
| EE (kcal/min) | 1.38 | ± | 0.24 | 1.38 | ± | 0.16 | 0.00 | -0.51 | - | 0.51 | 0.997 | 0.20 |
| FAT (mg/min) | 73.8 | ± | 26.3 | 85.4 | ± | 30.4 | -11.62 | -58.39 | - | 35.16 | 0.070 | 0.60 |
| CHO (mg/min) | 170.2 | ± | 83.0 | 141.0 | ± | 62.4 | 29.19 | -100.27 | - | 158.66 | 0.097 | 0.55 |
| P_ET_O_2_ (mmHg) | 104.3 | ± | 4.4 | 112.8 | ± | 4.7 | -8.50 | -17.26 | - | 0.25 | 0.000 | 0.19 |
| P_ET_CO_2_ (mmHg) | 36.7 | ± | 2.7 | 26.9 | ± | 4.5 | 9.75 | 2.81 | - | 16.70 | 0.000 | 0.13 |
| Values are means ± standard deviation (SD). VO_2_, oxygen uptake; VCO_2_, carbon dioxide production; RER, respiratory exchange ratio; Rf, respiratory frequency; V_E_, ventilation; V_T_, tidal volume; V_E_/VO_2_, ventilatory equivalent for O_2_; V_E_/VCO_2_, ventilatory equivalent for CO_2_; F_I_O_2_, inspiratory O_2_ fraction; F_E_O_2_, expiratory O_2_ fraction; F_I_CO_2_, inspiratory CO_2_ fraction; F_E_CO_2_, expiratory CO_2_ fraction; EE, energy expenditure; FAT, fatty acid oxidation; CHO, carbohydrate oxidation; P_ET_O_2_, end-tidal O_2_ pressure; P_ET_CO_2_, end-tidal CO_2_ pressure; CCC; concordance correlation coefficient; (n=16). | | | | | | | | | | | | |
|  |  |  |  |  |  |  |  |  |  |  |  |  |
|  |  |  |  |  |  |  |  |  |  |  |  |  |
|  |  |  |  |  |  |  |  |  |  |  |  |  |
|  |  |  |  |  |  |  |  |  |  |  |  |  |
|  |  |  |  |  |  |  |  |  |  |  |  |  |

| **Table 2 continuation.** Accuracy of COSMED K5 (Mixing Chamber) compared to Vyntus during resting measurements | | | | | | | | | | | | |
| --- | --- | --- | --- | --- | --- | --- | --- | --- | --- | --- | --- | --- |
|  | **Vyntus** | | | **COSMED K5 (Mix)** | | | **Mean differences** | **Limits of agreement** | | | **T-Test P-Value** | **CCC** |
|  | **Mean** | **±** | **SD** | **Mean** | **±** | **SD** |  | **Lower** | - | **Upper** |  |  |
| VO_2_ (mL/min) | 275 | ± | 47 | 312 | ± | 44 | -36.78 | -150.55 | - | 76.99 | 0.023 | 0.16 |
| VCO_2_ (mL/min) | 231 | ± | 46 | 224 | ± | 30 | 6.94 | -89.17 | - | 103.05 | 0.580 | 0.22 |
| RER | 0.83 | ± | 0.06 | 0.72 | ± | 0.04 | 0.11 | 0.03 | - | 0.20 | 0.000 | 0.13 |
| Rf (1/min) | 14.4 | ± | 2.3 | 13.9 | ± | 3.0 | 0.42 | -3.05 | - | 3.88 | 0.362 | 1.01 |
| V_T_ (L) | 0.55 | ± | 0.08 | 0.65 | ± | 0.25 | -0.10 | -0.55 | - | 0.35 | 0.092 | 0.46 |
| V_E_ (L/min) | 8.3 | ± | 1.4 | 8.4 | ± | 1.2 | -0.06 | -3.25 | - | 3.14 | 0.892 | 0.23 |
| V_E_/VO_2_ | 30.5 | ± | 4.1 | 26.9 | ± | 2.1 | 3.58 | -1.56 | - | 8.72 | 0.000 | 0.42 |
| V_E_/VCO_2_ | 36.5 | ± | 4.5 | 37.5 | ± | 3.5 | -1.05 | -6.14 | - | 4.04 | 0.126 | 0.86 |
| F_I_O_2_ (%) | 20.3 | ± | 0.3 | 20.9 | ± | 0.0 | -0.60 | -1.24 | - | 0.03 | 0.000 | 0.00 |
| F_E_O_2_ (%) | 16.36 | ± | 0.43 | 16.51 | ± | 0.36 | -0.15 | -0.70 | - | 0.39 | 0.044 | 0.76 |
| F_I_CO_2_ (%) | 0.47 | ± | 0.24 | 0.05 | ± | 0.01 | 0.42 | -0.05 | - | 0.89 | 0.000 | 0.00 |
| F_E_CO_2_ (%) | 3.86 | ± | 0.27 | 3.42 | ± | 0.31 | 0.44 | 0.02 | - | 0.87 | 0.000 | 0.34 |
| EE (kcal/min) | 1.38 | ± | 0.24 | 1.52 | ± | 0.21 | -0.14 | -0.71 | - | 0.43 | 0.071 | 0.17 |
| FAT (mg/min) | 73.8 | ± | 26.3 | 147.9 | ± | 32.0 | -74.13 | -126.52 | - | -21.74 | 0.000 | 0.14 |
| CHO (mg/min) | 170.2 | ± | 83.0 | 19.7 | ± | 51.8 | 150.45 | 27.54 | - | 273.36 | 0.000 | 0.17 |
| P_ET_O_2_ (mmHg) | 104.3 | ± | 4.4 | 113.7 | ± | 2.5 | -9.39 | -15.80 | - | -2.98 | 0.000 | 0.12 |
| P_ET_CO_2_ (mmHg) | 36.7 | ± | 2.7 | 23.5 | ± | 2.2 | 13.14 | 7.97 | - | 18.30 | 0.000 | 0.03 |
| Values are means ± standard deviation (SD). VO_2_, oxygen uptake; VCO_2_, carbon dioxide production; RER, respiratory exchange ratio; Rf, respiratory frequency; V_E_, ventilation; V_T_, tidal volume; V_E_/VO_2_, ventilatory equivalent for O_2_; V_E_/VCO_2_, ventilatory equivalent for CO_2_; F_I_O_2_, inspiratory O_2_ fraction; F_E_O_2_, expiratory O_2_ fraction; F_I_CO_2_, inspiratory CO_2_ fraction; F_E_CO_2_, expiratory CO_2_ fraction; EE, energy expenditure; FAT, fatty acid oxidation; CHO, carbohydrate oxidation; P_ET_O_2_, end-tidal O_2_ pressure; P_ET_CO_2_, end-tidal CO_2_ pressure; CCC; concordance correlation coefficient; (n=16). | | | | | | | | | | | | |
|  |  |  |  |  |  |  |  |  |  |  |  |  |
|  |  |  |  |  |  |  |  |  |  |  |  |  |
|  |  |  |  |  |  |  |  |  |  |  |  |  |
|  |  |  |  |  |  |  |  |  |  |  |  |  |
|  |  |  |  |  |  |  |  |  |  |  |  |  |
