# Supplementary material for: Accuracy and Precision of the COSMED K5 Portable Analyser
Source: Front Physiol. 2018 Dec 21;9:1764. doi: 10.3389/fphys.2018.01764 (PMC6308190; doi:10.3389/fphys.2018.01764)
Supplement: Supplementary file 3 [file Table_3.docx]

| **Table 3.** Accuracy of COSMED K5 (Breath by Breath) compared to Vyntus during exercise at low and moderate intensity | | | | | | | | | | | | | |
| --- | --- | --- | --- | --- | --- | --- | --- | --- | --- | --- | --- | --- | --- |
|  |  | **Vyntus** | | | **COSMED K5 (BxB)** | | | **Mean differences** | **Limits of agreement** | | | **T-Test P-Value** |  |
|  | **LOAD (W)** | **Mean** | **±** | **SD** | **Mean** | **±** | **SD** |  | **Lower** | **-** | **Upper** |  | **CCC** |
| VO_2_ (mL/min) | 60W | 1,292 | ± | 106 | 1,244 | ± | 107 | 48.07 | -154.86 | - | 251.01 | 0.083 | 0.48 |
| VCO_2_ (mL/min) | 60W | 1,101 | ± | 98 | 1,082 | ± | 119 | 18.95 | -176.61 | - | 214.50 | 0.459 | 0.57 |
| RER | 60W | 0.85 | ± | 0.04 | 0.87 | ± | 0.04 | -0.02 | -0.12 | - | 0.09 | 0.254 | 0.08 |
| Rf (1/min) | 60W | 23.5 | ± | 5.1 | 24.0 | ± | 4.0 | -0.49 | -6.89 | - | 5.92 | 0.559 | 0.74 |
| V_T_ (L) | 60W | 1.44 | ± | 0.37 | 1.39 | ± | 0.22 | 0.05 | -0.47 | - | 0.57 | 0.464 | 0.60 |
| V_E_ (L/min) | 60W | 32.4 | ± | 4.2 | 32.4 | ± | 3.2 | -0.06 | -8.55 | - | 8.43 | 0.956 | 0.33 |
| V_E_/VO_2_ | 60W | 25.1 | ± | 2.7 | 26.1 | ± | 1.6 | -1.04 | -5.56 | - | 3.48 | 0.092 | 0.41 |
| V_E_/VCO_2_ | 60W | 29.4 | ± | 2.5 | 30.1 | ± | 1.6 | -0.68 | -4.77 | - | 3.41 | 0.210 | 0.49 |
| F_I_O_2_ (%) | 60W | 20.8 | ± | 0.1 | 20.9 | ± | 0.0 | -0.15 | -0.27 | - | -0.04 | 0.000 | 0.00 |
| F_E_O_2_ (%) | 60W | 15.87 | ± | 0.53 | 16.22 | ± | 0.27 | -0.35 | -1.19 | - | 0.50 | 0.006 | 0.35 |
| F_I_CO_2_ (%) | 60W | 0.13 | ± | 0.04 | 0.06 | ± | 0.01 | 0.07 | -0.02 | - | 0.16 | 0.000 | 0.00 |
| F_E_CO_2_ (%) | 60W | 4.41 | ± | 0.39 | 4.24 | ± | 0.21 | 0.16 | -0.47 | - | 0.80 | 0.059 | 0.42 |
| EE (kcal/min) | 60W | 6.49 | ± | 0.53 | 6.27 | ± | 0.56 | 0.22 | -0.78 | - | 1.21 | 0.109 | 0.52 |
| FAT (mg/min) | 60W | 316.5 | ± | 90.1 | 267.3 | ± | 85.7 | 49.23 | -206.87 | - | 305.34 | 0.153 | -0.09 |
| CHO (mg/min) | 60W | 880.5 | ± | 228.8 | 948.7 | ± | 274.4 | -68.19 | -696.22 | - | 559.84 | 0.408 | 0.19 |
| P_ET_O_2_ (mmHg) | 60W | 99.3 | ± | 4.3 | 100.5 | ± | 2.7 | -1.23 | -8.18 | - | 5.73 | 0.187 | 0.48 |
| P_ET_CO_2_ (mmHg) | 60W | 39.6 | ± | 2.6 | 39.7 | ± | 2.2 | -0.07 | -4.59 | - | 4.45 | 0.905 | 0.55 |
|  |  |  |  |  |  |  |  |  |  |  |  |  |  |
| VO_2_ (mL/min) | Moderate Load | 2,375 | ± | 220 | 2,218 | ± | 226 | 157.76 | -132.40 | - | 447.92 | 0.001 | 0.62 |
| VCO_2_ (mL/min) | Moderate Load | 2,371 | ± | 317 | 2,207 | ± | 273 | 163.31 | -146.76 | - | 473.38 | 0.001 | 0.74 |
| RER | Moderate Load | 1.00 | ± | 0.07 | 1.00 | ± | 0.08 | 0.00 | -0.12 | - | 0.12 | 0.967 | 0.69 |
| Rf (1/min) | Moderate Load | 33.5 | ± | 7.7 | 33.1 | ± | 7.9 | 0.49 | -3.97 | - | 4.96 | 0.399 | 0.96 |
| V_T_ (L) | Moderate Load | 2.13 | ± | 0.37 | 2.07 | ± | 0.34 | 0.06 | -0.31 | - | 0.42 | 0.247 | 0.85 |
| V_E_ (L/min) | Moderate Load | 68.8 | ± | 11.8 | 66.7 | ± | 11.0 | 2.08 | -5.55 | - | 9.72 | 0.049 | 0.93 |
| V_E_/VO_2_ | Moderate Load | 29.0 | ± | 3.8 | 30.2 | ± | 4.4 | -1.22 | -4.55 | - | 2.11 | 0.012 | 0.88 |
| V_E_/VCO_2_ | Moderate Load | 29.0 | ± | 2.3 | 30.3 | ± | 3.3 | -1.27 | -4.66 | - | 2.11 | 0.010 | 0.74 |
| F_I_O_2_ (%) | Moderate Load | 20.8 | ± | 0.0 | 20.9 | ± | 0.0 | -0.13 | -0.21 | - | -0.04 | 0.000 | 0.00 |
| F_E_O_2_ (%) | Moderate Load | 16.39 | ± | 0.51 | 16.68 | ± | 0.55 | -0.29 | -0.71 | - | 0.14 | 0.000 | 0.80 |
| F_I_CO_2_ (%) | Moderate Load | 0.13 | ± | 0.04 | 0.06 | ± | 0.01 | 0.07 | -0.01 | - | 0.15 | 0.000 | 0.00 |
| F_E_CO_2_ (%) | Moderate Load | 4.48 | ± | 0.33 | 4.25 | ± | 0.45 | 0.23 | -0.25 | - | 0.70 | 0.002 | 0.70 |
| EE (kcal/min) | Moderate Load | 12.25 | ± | 1.15 | 11.44 | ± | 1.16 | 0.82 | -0.69 | - | 2.32 | 0.001 | 0.62 |
| FAT (mg/min) | Moderate Load | 108.1 | ± | 194.9 | 104.0 | ± | 152.6 | 4.13 | -212.40 | - | 220.65 | 0.883 | 0.80 |
| CHO (mg/min) | Moderate Load | 2,895.3 | ± | 599.1 | 2,694.9 | ± | 452.6 | 200.39 | -478.01 | - | 878.79 | 0.035 | 0.74 |
| P_ET_O_2_ (mmHg) | Moderate Load | 104.6 | ± | 4.1 | 104.2 | ± | 4.7 | 0.47 | -1.97 | - | 2.90 | 0.154 | 0.96 |
| P_ET_CO_2_ (mmHg) | Moderate Load | 39.3 | ± | 2.3 | 40.1 | ± | 3.9 | -0.79 | -4.84 | - | 3.26 | 0.147 | 0.77 |
| Values are means ± standard deviation (SD). VO_2_, oxygen uptake; VCO_2_, carbon dioxide production; RER, respiratory exchange ratio; Rf, respiratory frequency; V_E_, ventilation; V_T_, tidal volume; V_E_/VO_2_, ventilatory equivalent for O_2_; V_E_/VCO_2_, ventilatory equivalent for CO_2_; F_I_O_2_, inspiratory O_2_ fraction; F_E_O_2_, expiratory O_2_ fraction; F_I_CO_2_, inspiratory CO_2_ fraction; F_E_CO_2_, expiratory CO_2_ fraction; EE, energy expenditure; FAT, fatty acid oxidation; CHO, carbohydrate oxidation; P_ET_O_2_, end-tidal O_2_ pressure; P_ET_CO_2_, end-tidal CO_2_ pressure; Moderate Load, 130W in women, 160W in men, CCC; concordance correlation coefficient; (n=16). | | | | | | | | | | | | | |
|  |  |  |  |  |  |  |  |  |  |  |  |  |  |
|  |  |  |  |  |  |  |  |  |  |  |  |  |  |
|  |  |  |  |  |  |  |  |  |  |  |  |  |  |
|  |  |  |  |  |  |  |  |  |  |  |  |  |  |
|  |  |  |  |  |  |  |  |  |  |  |  |  |  |
|  |  |  |  |  |  |  |  |  |  |  |  |  |  |

| **Table 3 continuation.** Accuracy of COSMED K5 (Mixing Chamber) compared to Vyntus during exercise at low and moderate intensity | | | | | | | | | | | | | |
| --- | --- | --- | --- | --- | --- | --- | --- | --- | --- | --- | --- | --- | --- |
|  | **LOAD (W)** | **Vyntus** | | | **COSMED K5 (Mix)** | | | **Mean differences** | **Limits of agreement** | | | **T-Test P-Value** | **CCC** |
|  |  | **Mean** | **±** | **SD** | **Mean** | **±** | **SD** |  | **Lower** | **-** | **Upper** |  |  |
| VO_2_ (mL/min) | 60W | 1,292 | ± | 106 | 1,367 | ± | 137 | -75.12 | -328.45 | - | 178.20 | 0.035 | 0.46 |
| VCO_2_ (mL/min) | 60W | 1,101 | ± | 98 | 1,099 | ± | 131 | 1.83 | -269.79 | - | 273.46 | 0.959 | 0.32 |
| RER | 60W | 0.85 | ± | 0.04 | 0.80 | ± | 0.05 | 0.05 | -0.05 | - | 0.15 | 0.002 | 0.19 |
| Rf (1/min) | 60W | 23.5 | ± | 5.1 | 24.0 | ± | 4.8 | -0.46 | -6.54 | - | 5.63 | 0.566 | 0.92 |
| V_T_ (L) (btps) | 60W | 1.44 | ± | 0.37 | 1.42 | ± | 0.32 | 0.02 | -0.40 | - | 0.44 | 0.656 | 1.03 |
| V_E_ (L/min) | 60W | 32.4 | ± | 4.2 | 32.7 | ± | 3.8 | -0.34 | -8.69 | - | 8.01 | 0.755 | 0.49 |
| V_E_/VO_2_ | 60W | 25.1 | ± | 2.7 | 24.0 | ± | 1.9 | 1.09 | -1.64 | - | 3.81 | 0.007 | 0.82 |
| V_E_/VCO_2_ | 60W | 29.4 | ± | 2.5 | 29.9 | ± | 2.1 | -0.48 | -3.62 | - | 2.66 | 0.248 | 0.90 |
| F_I_O_2_ (%) | 60W | 20.8 | ± | 0.1 | 20.9 | ± | 0.0 | -0.15 | -0.27 | - | -0.04 | 0.000 | 0.00 |
| F_E_O_2_ (%) | 60W | 15.87 | ± | 0.53 | 15.87 | ± | 0.39 | 0.00 | -0.48 | - | 0.49 | 0.951 | 1.04 |
| F_I_CO_2_ (%) | 60W | 0.13 | ± | 0.04 | 0.06 | ± | 0.01 | 0.07 | -0.02 | - | 0.16 | 0.000 | -0.02 |
| F_E_CO_2_ (%) | 60W | 4.41 | ± | 0.39 | 4.28 | ± | 0.30 | 0.13 | -0.35 | - | 0.60 | 0.051 | 0.85 |
| EE (kcal/min) | 60W | 6.49 | ± | 0.53 | 6.79 | ± | 0.69 | -0.30 | -1.60 | - | 1.00 | 0.090 | 0.45 |
| FAT (mg/min) | 60W | 316.5 | ± | 90.1 | 446.9 | ± | 121.7 | -130.42 | -373.93 | - | 113.09 | 0.001 | 0.23 |
| CHO (mg/min) | 60W | 880.5 | ± | 228.8 | 629.8 | ± | 317.8 | 250.71 | -443.62 | - | 945.05 | 0.013 | 0.15 |
| P_ET_O_2_ (mmHg) | 60W | 99.3 | ± | 4.3 | 109.3 | ± | 2.7 | -9.98 | -14.56 | - | -5.39 | 0.000 | 0.16 |
| P_ET_CO_2_ (mmHg) | 60W | 39.6 | ± | 2.6 | 29.5 | ± | 2.0 | 10.15 | 7.24 | - | 13.06 | 0.000 | 0.08 |
|  |  |  |  |  |  |  |  |  |  |  |  |  |  |
| VO_2_ (mL/min) | Moderate Load | 2,375 | ± | 220 | 2,490 | ± | 241 | -114.70 | -389.06 | - | 159.66 | 0.005 | 0.77 |
| VCO_2_ (mL/min) | Moderate Load | 2,371 | ± | 317 | 2,364 | ± | 313 | 6.11 | -276.75 | - | 288.98 | 0.868 | 1.01 |
| RER | Moderate Load | 1.00 | ± | 0.07 | 0.95 | ± | 0.07 | 0.05 | -0.06 | - | 0.15 | 0.003 | 0.53 |
| Rf (1/min) | Moderate Load | 33.5 | ± | 7.7 | 32.6 | ± | 7.8 | 0.96 | -6.61 | - | 8.52 | 0.338 | 0.87 |
| V_T_ (L) (btps) | Moderate Load | 2.13 | ± | 0.37 | 2.13 | ± | 0.39 | -0.01 | -0.48 | - | 0.47 | 0.896 | 0.90 |
| V_E_ (L/min) | Moderate Load | 68.8 | ± | 11.8 | 67.5 | ± | 10.9 | 1.30 | -7.10 | - | 9.69 | 0.245 | 0.92 |
| V_E_/VO_2_ | Moderate Load | 29.0 | ± | 3.8 | 27.1 | ± | 3.5 | 1.83 | -1.93 | - | 5.60 | 0.002 | 0.62 |
| V_E_/VCO_2_ | Moderate Load | 29.0 | ± | 2.3 | 28.6 | ± | 2.8 | 0.41 | -2.83 | - | 3.65 | 0.334 | 0.63 |
| F_I_O_2_ (%) | Moderate Load | 20.8 | ± | 0.0 | 20.9 | ± | 0.0 | -0.13 | -0.22 | - | -0.04 | 0.000 | -0.01 |
| F_E_O_2_ (%) | Moderate Load | 16.39 | ± | 0.51 | 16.28 | ± | 0.51 | 0.12 | -0.36 | - | 0.59 | 0.073 | 0.80 |
| F_I_CO_2_ (%) | Moderate Load | 0.13 | ± | 0.04 | 0.06 | ± | 0.01 | 0.07 | -0.02 | - | 0.16 | 0.000 | -0.04 |
| F_E_CO_2_ (%) | Moderate Load | 4.48 | ± | 0.33 | 4.48 | ± | 0.39 | -0.01 | -0.46 | - | 0.44 | 0.925 | 0.67 |
| EE (kcal/min) | Moderate Load | 12.25 | ± | 1.15 | 12.75 | ± | 1.27 | -0.50 | -1.91 | - | 0.91 | 0.014 | 0.83 |
| FAT (mg/min) | Moderate Load | 108.1 | ± | 194.9 | 248.6 | ± | 218.6 | -140.46 | -448.08 | - | 167.16 | 0.003 | 0.75 |
| CHO (mg/min) | Moderate Load | 2,895.3 | ± | 599.1 | 2,670.9 | ± | 686.2 | 224.40 | -626.85 | - | 1,075.64 | 0.056 | 1.04 |
| P_ET_O_2_ (mmHg) | Moderate Load | 104.6 | ± | 4.1 | 112.0 | ± | 3.5 | -7.37 | -11.42 | - | -3.32 | 0.000 | 0.27 |
| P_ET_CO_2_ (mmHg) | Moderate Load | 39.3 | ± | 2.3 | 30.9 | ± | 2.7 | 8.47 | 5.17 | - | 11.77 | 0.000 | 0.11 |
| Values are means ± standard deviation (SD). VO_2_, oxygen uptake; VCO_2_, carbon dioxide production; RER, respiratory exchange ratio; Rf, respiratory frequency; V_E_, ventilation; V_T_, tidal volume; V_E_/VO_2_, ventilatory equivalent for O_2_; V_E_/VCO_2_, ventilatory equivalent for CO_2_; F_I_O_2_, inspiratory O_2_ fraction; F_E_O_2_, expiratory O_2_ fraction; F_I_CO_2_, inspiratory CO_2_ fraction; F_E_CO_2_, expiratory CO_2_ fraction; EE, energy expenditure; FAT, fatty acid oxidation; CHO, carbohydrate oxidation; P_ET_O_2_, end-tidal O_2_ pressure; P_ET_CO_2_, end-tidal CO_2_ pressure; Moderate Load, 130W in women, 160W in men, CCC; concordance correlation coefficient; (n=16). | | | | | | | | | | | | | |
|  |  |  |  |  |  |  |  |  |  |  |  |  |  |
|  |  |  |  |  |  |  |  |  |  |  |  |  |  |
|  |  |  |  |  |  |  |  |  |  |  |  |  |  |
|  |  |  |  |  |  |  |  |  |  |  |  |  |  |
|  |  |  |  |  |  |  |  |  |  |  |  |  |  |
|  |  |  |  |  |  |  |  |  |  |  |  |  |  |
