# Supplementary material for: Accuracy and Precision of the COSMED K5 Portable Analyser
Source: Front Physiol. 2018 Dec 21;9:1764. doi: 10.3389/fphys.2018.01764 (PMC6308190; doi:10.3389/fphys.2018.01764)
Supplement: Supplementary file 4 [file Table_4.docx]

| **Table 4.** Agreement between the Breath by Breath and Mixing Chamber of COSMED K5 during measurements at rest. | | | | | | | | | | | | |
| --- | --- | --- | --- | --- | --- | --- | --- | --- | --- | --- | --- | --- |
|  | **COSMED K5 (BxB)** | | | **COSMED K5 (Mix)** | | | **Mean differences** | **Limits of agreement** | | | **T-Test P-Value** | **CCC** |
|  | **Mean** | **±** | **SD** | **Mean** | **±** | **SD** |  | **Lower** | - | **Upper** |  |  |
| VO_2_ (mL/min) | 277 | ± | 33 | 312 | ± | 44 | -35.20 | -134.91 | - | 64.50 | 0.014 | 0.10 |
| VCO_2_ (mL/min) | 225 | ± | 25 | 224 | ± | 30 | 1.68 | -71.16 | - | 74.52 | 0.859 | 0.11 |
| RER | 0.82 | ± | 0.05 | 0.72 | ± | 0.04 | 0.10 | 0.02 | - | 0.17 | 0.000 | 0.20 |
| Rf (1/min) | 14.1 | ± | 2.3 | 13.9 | ± | 3.0 | 0.11 | -3.51 | - | 3.73 | 0.809 | 0.76 |
| V_T_ (L) | 0.57 | ± | 0.12 | 0.65 | ± | 0.25 | -0.08 | -0.48 | - | 0.31 | 0.118 | 0.41 |
| V_E_ (L/min) | 7.6 | ± | 0.8 | 8.4 | ± | 1.2 | -0.79 | -3.70 | - | 2.12 | 0.051 | -0.05 |
| V_E_/VO_2_ | 27.5 | ± | 2.2 | 26.9 | ± | 2.1 | 0.58 | -3.11 | - | 4.27 | 0.238 | 0.59 |
| V_E_/VCO_2_ | 33.7 | ± | 2.9 | 37.5 | ± | 3.5 | -3.79 | -9.69 | - | 2.11 | 0.000 | 0.33 |
| F_I_O_2_ (%) | 20.9 | ± | 0.0 | 20.9 | ± | 0.0 | 0.00 | -0.02 | - | 0.01 | 0.111 | 0.42 |
| F_E_O_2_ (%) | 16.51 | ± | 0.33 | 16.51 | ± | 0.36 | 0.00 | -0.55 | - | 0.56 | 0.957 | 0.66 |
| F_I_CO_2_ (%) | 0.05 | ± | 0.01 | 0.05 | ± | 0.01 | 0.00 | -0.01 | - | 0.02 | 0.111 | 0.42 |
| F_E_CO_2_ (%) | 3.77 | ± | 0.32 | 3.42 | ± | 0.31 | 0.35 | -0.20 | - | 0.91 | 0.000 | 0.36 |
| EE (kcal/min) | 1.38 | ± | 0.16 | 1.52 | ± | 0.21 | -0.14 | -0.63 | - | 0.34 | 0.039 | 0.09 |
| FAT (mg/min) | 85.4 | ± | 30.4 | 147.9 | ± | 32.0 | -62.52 | -119.49 | - | -5.54 | 0.000 | 0.19 |
| CHO (mg/min) | 141.0 | ± | 62.4 | 19.7 | ± | 51.8 | 121.26 | 41.28 | - | 201.23 | 0.000 | 0.23 |
| P_ET_O_2_ (mmHg) | 112.8 | ± | 4.7 | 113.7 | ± | 2.5 | -0.89 | -10.27 | - | 8.50 | 0.470 | 0.19 |
| P_ET_CO_2_ (mmHg) | 26.9 | ± | 4.5 | 23.5 | ± | 2.2 | 3.38 | -5.76 | - | 12.53 | 0.011 | 0.10 |
| Values are means ± standard deviation (SD). VO_2_, oxygen uptake; VCO_2_, carbon dioxide production; RER, respiratory exchange ratio; Rf, respiratory frequency; V_E_, ventilation; V_T_, tidal volume; V_E_/VO_2_, ventilatory equivalent for O_2_; V_E_/VCO_2_, ventilatory equivalent for CO_2_; F_I_O_2_, inspiratory O_2_ fraction; F_E_O_2_, expiratory O_2_ fraction; F_I_CO_2_, inspiratory CO_2_ fraction; F_E_CO_2_, expiratory CO_2_ fraction; EE, energy expenditure; FAT, fatty acid oxidation; CHO, carbohydrate oxidation; P_ET_O_2_, end-tidal O_2_ pressure; P_ET_CO_2_, end-tidal CO_2_ pressure; CCC; concordance correlation coefficient; (n=16). | | | | | | | | | | | | |
|  |  |  |  |  |  |  |  |  |  |  |  |  |
|  |  |  |  |  |  |  |  |  |  |  |  |  |
|  |  |  |  |  |  |  |  |  |  |  |  |  |
|  |  |  |  |  |  |  |  |  |  |  |  |  |
|  |  |  |  |  |  |  |  |  |  |  |  |  |
