# Supplementary material for: Accuracy and Precision of the COSMED K5 Portable Analyser
Source: Front Physiol. 2018 Dec 21;9:1764. doi: 10.3389/fphys.2018.01764 (PMC6308190; doi:10.3389/fphys.2018.01764)
Supplement: Supplementary file 5 [file Table_5.docx]

| **Table 5.** Agreement between the Breath by Breath and Mixing Chamber modes of COSMED K5 during exercise at low and moderate intensity. | | | | | | | | | | | | | |
| --- | --- | --- | --- | --- | --- | --- | --- | --- | --- | --- | --- | --- | --- |
|  | **LOAD (W)** | **COSMED K5 (BxB)** | | | **COSMED K5 (Mix)** | | | **Mean differences** | **Limits of agreement** | | | **T-Test P-Value** | **CCC** |
|  |  | **Mean** | **±** | **SD** | **Mean** | **±** | **SD** |  | **Lower** | **-** | **Upper** |  |  |
| VO_2_ (mL/min) | 60W | 1,244 | ± | 107 | 1,367 | ± | 137 | -123.20 | -375.34 | - | 128.94 | 0.002 | 0.30 |
| VCO_2_ (mL/min) | 60W | 1,082 | ± | 119 | 1,099 | ± | 131 | -17.12 | -286.69 | - | 252.46 | 0.626 | 0.39 |
| RER | 60W | 0.87 | ± | 0.04 | 0.80 | ± | 0.05 | 0.07 | -0.04 | - | 0.17 | 0.000 | 0.13 |
| Rf (1/min) | 60W | 24.0 | ± | 4.0 | 24.0 | ± | 4.8 | 0.03 | -4.52 | - | 4.59 | 0.956 | 0.86 |
| V_T_ (L) | 60W | 1.39 | ± | 0.22 | 1.42 | ± | 0.32 | -0.03 | -0.45 | - | 0.40 | 0.642 | 0.68 |
| V_E_ (L/min) | 60W | 32.4 | ± | 3.2 | 32.7 | ± | 3.8 | -0.28 | -8.09 | - | 7.54 | 0.784 | 0.35 |
| V_E_/VO_2_ | 60W | 26.1 | ± | 1.6 | 24.0 | ± | 1.9 | 2.12 | -1.40 | - | 5.65 | 0.000 | 0.28 |
| V_E_/VCO_2_ | 60W | 30.1 | ± | 1.6 | 29.9 | ± | 2.1 | 0.20 | -2.63 | - | 3.03 | 0.581 | 0.69 |
| F_I_O_2_ (%) | 60W | 20.9 | ± | 0.0 | 20.9 | ± | 0.0 | 0.00 | -0.01 | - | 0.02 | 0.164 | 0.79 |
| F_E_O_2_ (%) | 60W | 16.22 | ± | 0.27 | 15.87 | ± | 0.39 | 0.35 | -0.29 | - | 1.00 | 0.001 | 0.34 |
| F_I_CO_2_ (%) | 60W | 0.06 | ± | 0.01 | 0.06 | ± | 0.01 | 0.00 | -0.02 | - | 0.01 | 0.164 | 0.79 |
| F_E_CO_2_ (%) | 60W | 4.24 | ± | 0.21 | 4.28 | ± | 0.30 | -0.04 | -0.46 | - | 0.39 | 0.513 | 0.65 |
| EE (kcal/min) | 60W | 6.27 | ± | 0.56 | 6.79 | ± | 0.69 | -0.52 | -1.81 | - | 0.77 | 0.007 | 0.33 |
| FAT (mg/min) | 60W | 267.3 | ± | 85.7 | 446.9 | ± | 121.7 | -179.66 | -439.89 | - | 80.58 | 0.000 | 0.08 |
| CHO (mg/min) | 60W | 948.7 | ± | 274.4 | 629.8 | ± | 317.8 | 318.90 | -402.94 | - | 1,040.75 | 0.003 | 0.15 |
| P_ET_O_2_ (mmHg) | 60W | 100.5 | ± | 2.7 | 109.3 | ± | 2.7 | -8.75 | -13.89 | - | -3.61 | 0.000 | 0.09 |
| P_ET_CO_2_ (mmHg) | 60W | 39.7 | ± | 2.2 | 29.5 | ± | 2.0 | 10.22 | 5.88 | - | 14.56 | 0.000 | 0.04 |
|  |  |  |  |  |  |  |  |  |  |  |  |  |  |
| VO_2_ (mL/min) | Moderate Load | 2,218 | ± | 226 | 2,490 | ± | 241 | -272.46 | -640.60 | - | 95.67 | 0.000 | 0.40 |
| VCO_2_ (mL/min) | Moderate Load | 2,207 | ± | 273 | 2,364 | ± | 313 | -157.20 | -473.62 | - | 159.22 | 0.001 | 0.74 |
| RER | Moderate Load | 1.00 | ± | 0.08 | 0.95 | ± | 0.07 | 0.05 | -0.05 | - | 0.15 | 0.002 | 0.62 |
| Rf (1/min) | Moderate Load | 33.1 | ± | 7.9 | 32.6 | ± | 7.8 | 0.46 | -5.41 | - | 6.34 | 0.547 | 0.93 |
| V_T_ (L) | Moderate Load | 2.07 | ± | 0.34 | 2.13 | ± | 0.39 | -0.06 | -0.46 | - | 0.33 | 0.221 | 0.83 |
| V_E_ (L/min) | Moderate Load | 66.7 | ± | 11.0 | 67.5 | ± | 10.9 | -0.79 | -6.04 | - | 4.47 | 0.260 | 0.97 |
| V_E_/VO_2_ | Moderate Load | 30.2 | ± | 4.4 | 27.1 | ± | 3.5 | 3.05 | -2.56 | - | 8.66 | 0.001 | 0.57 |
| V_E_/VCO_2_ | Moderate Load | 30.3 | ± | 3.3 | 28.6 | ± | 2.8 | 1.69 | -2.13 | - | 5.51 | 0.004 | 0.69 |
| F_I_O_2_ (%) | Moderate Load | 20.9 | ± | 0.0 | 20.9 | ± | 0.0 | 0.00 | -0.01 | - | 0.02 | 0.164 | 0.79 |
| F_E_O_2_ (%) | Moderate Load | 16.68 | ± | 0.55 | 16.28 | ± | 0.51 | 0.40 | -0.25 | - | 1.06 | 0.000 | 0.62 |
| F_I_CO_2_ (%) | Moderate Load | 0.06 | ± | 0.01 | 0.06 | ± | 0.01 | 0.00 | -0.02 | - | 0.01 | 0.164 | 0.79 |
| F_E_CO_2_ (%) | Moderate Load | 4.25 | ± | 0.45 | 4.48 | ± | 0.39 | -0.23 | -0.76 | - | 0.29 | 0.003 | 0.69 |
| EE (kcal/min) | Moderate Load | 11.44 | ± | 1.16 | 12.75 | ± | 1.27 | -1.32 | -3.24 | - | 0.60 | 0.000 | 0.42 |
| FAT (mg/min) | Moderate Load | 104.0 | ± | 152.6 | 248.6 | ± | 218.6 | -144.58 | -397.07 | - | 107.90 | 0.000 | 0.59 |
| CHO (mg/min) | Moderate Load | 2,694.9 | ± | 452.6 | 2,670.9 | ± | 686.2 | 24.01 | -820.70 | - | 868.72 | 0.827 | 0.72 |
| P_ET_O_2_ (mmHg) | Moderate Load | 104.2 | ± | 4.7 | 112.0 | ± | 3.5 | -7.83 | -12.29 | - | -3.38 | 0.000 | 0.30 |
| P_ET_CO_2_ (mmHg) | Moderate Load | 40.1 | ± | 3.9 | 30.9 | ± | 2.7 | 9.26 | 4.97 | - | 13.55 | 0.000 | 0.17 |
| Values are means ± standard deviation (SD). VO_2_, oxygen uptake; VCO_2_, carbon dioxide production; RER, respiratory exchange ratio; Rf, respiratory frequency; V_E_, ventilation; V_T_, tidal volume; V_E_/VO_2_, ventilatory equivalent for O_2_; V_E_/VCO_2_, ventilatory equivalent for CO_2_; F_I_O_2_, inspiratory O_2_ fraction; F_E_O_2_, expiratory O_2_ fraction; F_I_CO_2_, inspiratory CO_2_ fraction; F_E_CO_2_, expiratory CO_2_ fraction; EE, energy expenditure; FAT, fatty acid oxidation; CHO, carbohydrate oxidation; P_ET_O_2_, end-tidal O_2_ pressure; P_ET_CO_2_, end-tidal CO_2_ pressure; Moderate Load, 130W in women, 160W in men, CCC; concordance correlation coefficient; (n=16). | | | | | | | | | | | | | |
|  |  |  |  |  |  |  |  |  |  |  |  |  |  |
|  |  |  |  |  |  |  |  |  |  |  |  |  |  |
|  |  |  |  |  |  |  |  |  |  |  |  |  |  |
|  |  |  |  |  |  |  |  |  |  |  |  |  |  |
|  |  |  |  |  |  |  |  |  |  |  |  |  |  |
|  |  |  |  |  |  |  |  |  |  |  |  |  |  |
