# Supplementary material for: Accuracy and Precision of the COSMED K5 Portable Analyser
Source: Front Physiol. 2018 Dec 21;9:1764. doi: 10.3389/fphys.2018.01764 (PMC6308190; doi:10.3389/fphys.2018.01764)
Supplement: Supplementary file 6 [file Table_6.docx]

| **Table 6.** Precision of K5 during prolonged field operation in mixing chamber mode | | | | | | | | | | | | |
| --- | --- | --- | --- | --- | --- | --- | --- | --- | --- | --- | --- | --- |
|  | **1st measurement** | | | **2nd measurement** | | | **CV** | **95% CI** | | | **CCC** | **T-Test P-Value** |
|  | **Mean** | **±** | **SD** | **Mean** | **±** | **SD** |  |  |  |  |  |  |
| Body Mass Pre (kg) | 73.4 | ± | 10.5 | 74.0 | ± | 10.8 | 0.97 | 0.70 | - | 1.51 | 1.00 | 0.014 |
| Body Mass Post (kg) | 72.9 | ± | 10.4 | 73.4 | ± | 10.7 | 1.02 | 0.73 | - | 1.58 | 0.99 | 0.093 |
| Difference in Body Mass (kg) | -0.5 | ± | 0.3 | -0.6 | ± | 0.3 | 72.05 | 43.45 | - | 167.42 | 0.40 | 0.139 |
| VO_2_ (mL/min) | 1,193 | ± | 223 | 1,220 | ± | 248 | 4.42 | 3.18 | - | 6.85 | 0.95 | 0.180 |
| VO_2_/kg Pre (mL/min/kg) | 16.3 | ± | 2.4 | 16.5 | ± | 2.7 | 4.59 | 3.30 | - | 7.12 | 0.91 | 0.402 |
| VO_2_/kg Post (mL/min/kg) | 16.4 | ± | 2.4 | 16.7 | ± | 2.7 | 4.68 | 3.36 | - | 7.26 | 0.90 | 0.353 |
| VCO_2_ (mL/min) | 1,000 | ± | 212 | 1,033 | ± | 212 | 6.23 | 4.47 | - | 9.66 | 0.92 | 0.130 |
| RER | 0.84 | ± | 0.04 | 0.85 | ± | 0.03 | 3.36 | 2.41 | - | 5.20 | 0.41 | 0.269 |
| Rf (1/min) | 27.1 | ± | 4.9 | 27.8 | ± | 5.0 | 3.88 | 2.78 | - | 6.01 | 0.95 | 0.088 |
| V_E_ (L/min) | 31.2 | ± | 5.9 | 32.5 | ± | 6.3 | 6.86 | 4.91 | - | 10.62 | 0.89 | 0.079 |
| V_T_ (L) | 1.18 | ± | 0.25 | 1.20 | ± | 0.25 | 6.41 | 4.58 | - | 9.91 | 0.91 | 0.581 |
| V_E_/VO_2_ | 26.2 | ± | 1.8 | 26.7 | ± | 2.5 | 3.91 | 2.81 | - | 6.06 | 0.77 | 0.171 |
| V_E_/VCO_2_ | 31.4 | ± | 2.7 | 31.6 | ± | 3.0 | 3.39 | 2.44 | - | 5.25 | 0.85 | 0.691 |
| F_I_O_2_ | 20.9 | ± | 0.0 | 20.9 | ± | 0.0 | 0.01 | 0.01 | - | 0.01 | 0.63 | 0.336 |
| F_E_O_2_ | 16.49 | ± | 0.33 | 16.55 | ± | 0.42 | 0.95 | 0.68 | - | 1.46 | 0.82 | 0.311 |
| F_I_CO_2_ | 0.04 | ± | 0.00 | 0.04 | ± | 0.00 | 4.28 | 3.06 | - | 6.60 | 0.63 | 0.336 |
| F_E_CO_2_ | 3.88 | ± | 0.34 | 3.87 | ± | 0.38 | 3.35 | 2.41 | - | 5.19 | 0.87 | 0.843 |
| EE (kcal/min) | 5.97 | ± | 1.14 | 6.12 | ± | 1.24 | 4.64 | 3.33 | - | 7.19 | 0.95 | 0.154 |
| FAT (mg/min) | 320.2 | ± | 78.6 | 310.1 | ± | 90.2 | 16.23 | 11.58 | - | 25.57 | 0.57 | 0.635 |
| CHO (mg/min) | 737.6 | ± | 304.0 | 801.6 | ± | 230.6 | 24.51 | 17.20 | - | 39.23 | 0.67 | 0.281 |
| TEE (kcal) | 829 | ± | 117 | 849 | ± | 131 | 4.53 | 3.25 | - | 7.02 | 0.91 | 0.148 |
| TEE/km | 63.9 | ± | 9.2 | 65.4 | ± | 10.2 | 4.45 | 3.19 | - | 6.89 | 0.91 | 0.165 |
| TEE/kg | 11.3 | ± | 1.0 | 11.5 | ± | 1.3 | 4.61 | 3.31 | - | 7.14 | 0.78 | 0.336 |
| TEE/km/kg | 0.87 | ± | 0.08 | 0.89 | ± | 0.10 | 4.55 | 3.26 | - | 7.05 | 0.79 | 0.370 |
| HR (bpm) | 105.2 | ± | 14.0 | 99.6 | ± | 14.2 | 6.96 | 5.00 | - | 10.81 | 0.76 | 0.028 |
| GPS Speed (km/h) | 5.5 | ± | 0.5 | 5.5 | ± | 0.5 | 2.71 | 1.95 | - | 4.20 | 0.91 | 0.776 |
| Total GPS distance (m) | 12,967 | ± | 72 | 12,983 | ± | 57 | 0.40 | 0.29 | - | 0.62 | 0.35 | 0.436 |
| CV, coefficient of variation; CCC, concordance correlation coefficient; VO_2_, oxygen uptake; VCO_2_, carbon dioxide production; RER, respiratory exchange ratio; Rf, respiratory frequency; V_E_, ventilation; V_T_, tidal volume; V_E_/VO_2_, ventilatory equivalent for O_2_; V_E_/VCO_2_, ventilatory equivalent for CO_2_; F_I_O_2_, inspiratory O_2_ fraction; F_E_O_2_, expiratory O_2_ fraction; F_I_CO_2_, inspiratory CO_2_ fraction; F_E_CO_2_, expiratory CO_2_ fraction, EE, energy expenditure; FAT, fatty acid oxidation; CHO, carbohydrate oxidation; TEE, total energy expenditure; HR, heart rate; (n=14). | | | | | | | | | | | | |
|  |  |  |  |  |  |  |  |  |  |  |  |  |
|  |  |  |  |  |  |  |  |  |  |  |  |  |
|  |  |  |  |  |  |  |  |  |  |  |  |  |
