# Supplementary material for: Accuracy and Precision of the COSMED K5 Portable Analyser
Source: Front Physiol. 2018 Dec 21;9:1764. doi: 10.3389/fphys.2018.01764 (PMC6308190; doi:10.3389/fphys.2018.01764)
Supplement: Supplementary file 7 [file Table_7.docx]

| **Table 7.** Environmental conditions during field tests. | | | | | | | | |
| --- | --- | --- | --- | --- | --- | --- | --- | --- |
|  |  | **1st measurement** | | | **2nd measurement** | | | **P-Value** |
|  |  | **Mean** | **±** | **SD** | **Mean** | **±** | **SD** |  |
| Ambient  Temperature (ºC) | AVG Temp. (ºC) | 22.1 | ± | 3.1 | 22.4 | ± | 2.3 | 0.726 |
|  | Start Temp. (ºC) | 22.7 | ± | 3.5 | 23.0 | ± | 3.5 | 0.833 |
|  | Finish Temp. (ºC) | 20.8 | ± | 2.2 | 21.6 | ± | 3.8 | 0.495 |
|  | Diff. Ambient Temp. (ºC) | -1.9 | ± | 2.8 | -1.4 | ± | 3.9 | 0.715 |
| Ambient  Relative Humidity  (RH) | AVG RH Ambient (%) | 61.7 | ± | 8.7 | 63.5 | ± | 9.1 | 0.505 |
|  | Start RH (%) | 61.4 | ± | 10.3 | 63.1 | ± | 13.9 | 0.666 |
|  | Finish RH (%) | 66.5 | ± | 6.5 | 65.8 | ± | 14.5 | 0.859 |
|  | Diff. RH (%) | 5.1 | ± | 8.4 | 2.6 | ± | 18.5 | 0.636 |
| Barometric  Pressure (BP) | AVG BP (mmHg) | 765.8 | ± | 2.9 | 764.4 | ± | 3.3 | 0.308 |
|  | Start BP (mmHg) | 766.1 | ± | 3.2 | 764.7 | ± | 3.2 | 0.342 |
|  | Finish BP (mmHg) | 766.1 | ± | 2.9 | 764.6 | ± | 3.2 | 0.319 |
|  | Diff. BP (mmHg) | -0.1 | ± | 0.9 | -0.1 | ± | 0.7 | 1.000 |
